# Prevalence and risk factors of diabetes among adults aged 45 years or older in China: A national cross‐sectional study

**DOI:** 10.1002/edm2.265

**Published:** 2021-05-24

**Authors:** Anying Bai, Jing Tao, Liyuan Tao, Jue Liu

**Affiliations:** ^1^ School of Public Health Peking University Beijing China; ^2^ College of Rehabilitation Medicine Fujian University of Traditional Chinese Medicine Fujian China; ^3^ Research Center of Clinical Epidemiology Peking University Third Hospital Beijing China

**Keywords:** awareness, CHARLS, diabetes, pre‐diabetes, prevalence

## Abstract

**Background:**

Although there is preponderance of literature on disease burden of diabetes in developed countries, limited investigations have been conducted in less developed regions including China. This study aimed to explore the current prevalence and risk factors for diabetes, pre‐diabetes, awareness, treatment and control of diabetes in China.

**Methods:**

We included 12,458 adults from the China Health and Retirement Longitudinal Study. We estimated prevalence of diabetes and pre‐diabetes in the overall sample and by socio‐demographics. Bivariate associations of diabetes, pre‐diabetes, awareness, control and treatment of diabetes with health and function measures were evaluated by chi‐squared test and multivariate logistic regression analysis.

**Results:**

We found that the prevalence of diabetes and pre‐diabetes was 13.21% and 25.16%. The prevalence of diabetes increased with advanced age (12.37%, 15.98% and 16.52% among persons who were 45 to 55, 55 to 65 and ≥65 years old, respectively), educational background (14.52%, 15.52% and 15.58% among persons who were illiterate, had primary education and had secondary or above education) and weight (8.18%, 17.05% and 22.54% among persons with a body mass index of 18.5 to 24.9, 25.0 to 29.9 and ≥30.0, respectively). The prevalence of diabetes was higher among urban residents than among rural residents (19.04% vs. 12.85%). We also observed that aged between 55 and 65 years, obesity, history of hypertension and coronary heart disease, and inactivity were significant risk factors of awareness of diabetes.

**Conclusion:**

Our results indicated that diabetes is high prevalent in adults aged 45 years or above in China. The potentially modifiable risk factors should be further studied to develop interventions and strategies aimed at prevention and treatment of diabetes among middle‐aged and older Chinese adults.

## Graphical abstract

We included 12,458 adults from the China Health and Retirement Longitudinal Study. We estimated prevalence of diabetes and pre‐diabetes in the overall sample and by socio‐demographics. Bivariate associations of diabetes, pre‐diabetes, awareness, control and treatment of diabetes with health and function measures were evaluated by chi‐squared test and multivariate logistic regression analysis. Our results indicated that diabetes is high prevalent in adults aged 45 year or above in China. The potentially modifiable risk factors should be further studied to develop interventions and strategies aimed at prevention and treatment of diabetes among middle aged and older Chinese adults.

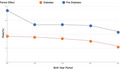

## 1 INTRODUCTION

Diabetes is one of the fastest‐growing chronic diseases in the world. In 2017, approximately 425 million adults worldwide had the condition, and about half of these cases had not been diagnosed. In China, the number of diabetic patients is estimated to increase from 20.8 million in 2000 to 42.3 million in 2030.^1^ The increase is thought to be the most rapid worldwide, and it might be related to China's recent rapid economic development and urbanization, which are contributing to socioeconomic and epidemiological transition. Previous research has shown that non‐communicable diseases are initially more common in population subgroups of high socioeconomic status (SES) and then, with increasing development, become more common in lower SES groups.^2^ However, these evidences are less shown in developing countries.

Studies have indicated that the grim situation regarding diabetes could be mitigated through appropriate management and education. However, diabetes awareness is low, especially in developing countries, and blood glucose control in particular remains a challenge for healthcare providers. In China, less than one‐third (30.1%) of diabetic patients were aware of their disease condition,^3^ and this may affect the estimate of diabetes prevalence. Research has found that adequate glycemic control can significantly decrease the risk of diabetes‐related complications, causing a delay in disease progression. Basic information about the prevalence, treatment and diabetic educational condition should be analysed to provide evidence for further prevention. Although previous studies have examined the prevalence of awareness, treatment and control of diabetes in different populations, the results were inconsistent due to the discrepancy in geographic regions and diagnosis criteria. Moreover, many researches were reported based on self‐reports. In addition, diabetes is a major risk factor for cardiovascular disease, which has become the leading cause of death in China. IDF reported that over 10% of health costs in China could be related to diabetes.^4^ It is known that social and economic factors such as income, educational level, occupation, levels of physical activity, being overweight or obese, health behaviours, living conditions and other demographic factors are strong influence factors of pre‐diabetes and diabetes,^5^, ^6^ while the physiological or genetic factors linking diabetes and cardiovascular disease are understudied. Therefore, we used data from a nationally representative cohort of middle‐aged and older adults in China to explore the prevalence and demographic as well as physiological risk factors of pre‐diabetes, diabetes, awareness, control and treatment of diabetes. More attention should be given to implement preventive strategy and interventions to improve the overall management of diabetes among community‐dwelling elders in China.

## 2 MATERIAL AND METHODS

### 2.1 Data source and study population

We use data collected from the survey of the China Health and Retirement Longitudinal Study (CHARLS), a national survey representative of the middle‐aged and elderly population (45 years old and above) in China. CHARLS adopted a four‐stage stratified cluster sampling to recruit participants. In the first stage, 150 county‐level units from 28 provinces were selected to provide a mix of urban and rural settings, with a wide variation in the level of economic development. The second stage randomly chose 939 primary sampling units (PSUs) (470 villages and 469 communities) from the above county‐level units. All the dwellings in each selected PSU were outlined on Google Earth maps using the CHARLS‐GIS software, which was specifically designed for the study. The third stage randomly selected 24 mapped households from each PSU. The last stage randomly selected 1 adult aged 45 years and older from each household.^7^ Finally, a total of 21,097 respondents were successfully enrolled in 2015. Of these respondents, cross‐section blood samples of 13,013 individuals were collected with a response rate of 64.15%, and our final analysis included 12,458 individuals who have complete information on demographic characteristics, physical function and clinical measures. The original CHARLS was approved by the Ethical Review Committee at Peking University (IRB00001052–11015). A ‘‘Letter to the Residents’’ leaflet was sent to each of the selected households. All participants provided written informed consent before the household survey. This study's data sets are publicly available at http: //charls.pku.edu.cn/en/page/data/2015‐charls‐wave4.

### 2.2 Measures of diabetes prevalence

#### 2.2.1 Definitions

In the present study, diabetes was defined as (1) a self‐reported previous diagnosis by healthcare professionals (2) fasting plasma glucose (FPG) >126 mg/dl and/or HbA1c >6.5%. The cut‐off points for diagnosis of diabetes were based on current recommendations from the American Diabetes Association.^8^ Pre‐diabetes was defined as 6.1 mmol/L<FPG <7.0 mmol/L.^9^ Awareness was defined as the proportion of individuals with self‐reported physician‐diagnosed diabetes among all participants with diabetes. Treatment was defined as the percentage of diabetic patients who had taken diabetic medication. Control was characterized as the rate of participants with an HbA1c level under 7.0% among diabetic patients who were treated with diabetic medications.

#### 2.2.2 Blood sample collection and quality control

The key advantage in using data collected from CHARLS is that blood samples were measured in the survey. Nearly two‐thirds blood samples of individuals were collected by medically trained staff from the China Center for Disease Prevention and Control (CDC). Participants were asked to fast overnight. After collection, plasma for glucose assay was separated from blood samples and stored at −20°C, and whole blood for HbA1C assay was stored immediately and during shipment at 4°C. All the blood samples were transported within 2 weeks to the CDC, where samples were placed at −80°C in a deep freezer before assay. Blood assays were performed at the Youanmen Center for Clinical Laboratory of Capital Medical University during February 2013 and June 2013. The laboratory used quality control samples daily during the testing of the CHARLS study samples, and all test results were within the target range (within two SDs of mean quality control concentrations). Glucose was measured using an enzymatic colorimetric test, and HbA1c was analysed using boronate affinity chromatography.

### 2.3 Other data collection

We sent two interviewers to each county‐level unit to interview about 72 households located in three communities. The interviewers were trained at Peking University by CHARLS staff members, and the interviews took place in respondents’ homes with the use of CAPI technology. The interviewers who conducted the county‐level interviews described above also carried equipment for and conducted measurements of health functioning and performance in respondents’ households. These included the anthropometric measurements of height, weight, waist circumference, lower right leg length and arm length, lung capacity, grip strength, speed of repeated chair stand, blood pressure, walking speed and balance tests.^7^

Our study included age and gender as individual attributes. We used two variables of socioeconomic status: level of education and residence area. We classified level of education as illiterate, primary education or above, and secondary education or above. All medical conditions including hypertension, diabetes and cardiac disease were diagnosed or assessed by a physician or using a self‐reported history of diagnosed condition. Disability was assessed by five activities of daily living (ADL) tasks (dressing, bathing, eating, getting out of bed and toileting). For each ADL task, participants were asked, ‘Do you have difficulty in’ performing the task? Those participants who responded, ‘I have difficulty but can still do it’, ‘Yes, I have difficulty and need help’ or ‘I cannot do it’ to one or more of the ADL tasks were considered having ADL disability.

Height and weight were measured and used to calculate body mass index (BMI: kg/m^2^) using standard formula. The survey also collected information on body mass index (BMI), which is the ratio of weight in kilograms to height in metres squared. We use BMI to identify whether respondents are underweight (BMI < 18.5), normal (18.5 < BMI < 24), overweight (24 < BMI < 28) and obese (BMI ≥ 28) based on the suggested categories for Asian populations. General questions about physical activity use in the ‘health status and functioning’ section of CHARLS questionnaire included the amount of time a person spent on different types of physical activities (PA, including vigorous activities, moderate activities and walking for at least 10 min continuously) in a usual week, and the PA scores was constructed by multiplying the number of days and the daily PA duration index for each activity based on the International Physical Activity Questionnaire (IPAQ). Physical inactivity was defined as not engaging in both low and moderate levels of activity, responding ‘no’ to both these questions. In this study, handgrip strength and FTSS test time were measured as physical performance variables by the trained evaluator. We measured muscle strength to the nearest 0.1 kg with an accurate handgrip dynamometer. The participants were asked to use their dominant hand twice with maximum effort and the mean of the two values was used for analysis. The FTSS test procedure was also carried out following standardized verbal instructions. After twice practicing the sit and stand, participants crossed their arms upon their chest and sat in the middle of a padded chair without arms with their back straight. They were instructed to fully straighten their legs when elevating, complete the sit between each stand and avoid bouncing off the chair when returning to the standing position. Participants were encouraged to stand up and sit down five times as quickly as possible, the time of the whole process from initial seated position to final seated position being recorded with a stopwatch.

Clinical measures were also used in this study. Blood pressure (BP; mmHg) was measured by an automatic BP monitor in the seated position. Three measurements, 45 seconds apart, were conducted, and the average was used. Fasting blood samples were collected by trained nurses in township hospital or a local office of the CDC. Blood‐based biomarkers included white blood cell count (10^3^/cm), platelets, (10^3^/cm), haemoglobin (g/dl), fasting glucose (mg/dl), low‐density lipoprotein cholesterol (mg/dl), high‐density lipoprotein cholesterol (mg/dl) and total cholesterol (mg/dl).

Participants were classified as having lower extremity functional limitation if they had difficulty performing any of the following tasks on a regular basis: getting up from a chair after sitting for a long period, climbing several flights of stairs without resting, or stooping, kneeling or crouching. Participants were considered having upper extremity functional limitation if they reported having difficulty in any of the following tasks: reaching or extending arms above shoulder level, lifting or carrying weights more than 5 kg or picking up a small coin from a table. For each task, participants were asked, ‘Do you have difficulty in’ performing the task? Those participants who responded, ‘I have difficulty but can still do it’, ‘Yes, I have difficulty and need help’ or ‘I cannot do it’ were considered having difficulty. Participants met criteria for exhaustion if they answered ‘A moderate amount of time; 3–4 days’ or ‘Most of the time’ to either of two questions from the modified Center for Epidemiological Studies‐Depression (CES‐D) scale: ‘I could not get going’ and ‘I felt everything I did was an effort’.

### 2.4 Statistical analysis

Age‐ and gender‐standardized prevalence of diabetes was calculated by the direct method using population census data of China in 2010. Characteristics of the study participants were depicted as the mean (95% CIs) for continuous variables and percentages (95% CIs) for categorical variables among all participants and in subgroups of sex. The χ^2^ test (for categorical variables) was used to analyse the differences in the socioeconomic characteristics, physical function and biomarkers between diabetic and pre‐diabetic individuals. The associations between awareness, control of glucose, treatment of diabetes (dependent variable) and independent variables were analysed using multivariate logistic regression analysis and reported as adjusted odds ratios (ORs) and 95% confidence intervals (95% CIs). Statistical significance was defined as a *p*‐value <.05. All analyses were performed using STATA software (version 14.0; Stata Corp LP. TX).

## 3 RESULTS

### 3.1 Basic characteristics

Table 1 summarizes and compares the general characteristics between male and female. The mean age of the study subjects was 60.66 years (SD 9.74 years). There were 5889 men (47.27%) and 6569 women (52.73%). The diastolic blood pressures and fasting glucose were not significantly different between different sex. All other selected characteristics showed significant differences between male and female (*p* < .05).

**TABLE 1:** General characteristics of the study population (*n* = 12,458).

Characteristics	Total (Mean ± SD)	Male (Mean ± SD)	Female (Mean ± SD)	*p*‐Value
*N*	12,458	5889	6569	<.001
Age (years)	60.66 ± 9.74	61.33 ± 9.74	60.05 ± 9.69	<.001
BMI (kg/m^2^)	24.97 ± 31.33	24.35 ± 30.92	25.53 ± 31.68	<.001
IPAQ score (Met/week)	61.49 ± 98.70	64.12 ± 101.94	59.12 ± 95.65	<.001
Grip strength (kg)	31.83 ± 12.33	38.23 ± 13.54	25.81 ± 6.92	<.001
Waist Circumference(cm)	85.50 ± 13.24	85.29 ± 13.36	85.68 ± 13.12	.028
Four‐metre usual gait speed (m/s)	1.34 ± 7.64	1.38±7.74	1.30 ± 7.55	
Systolic pressure (mmHg)	128.51 ± 19.92	129.56 ± 19.49	127.57 ± 20.26	.001
Diastolic pressure (mmHg)	75.47 ± 9.24	76.16 ± 9.60	74.85 ± 8.86	.068
WBC count, 10^3^/cm	5.99 ± 1.81	6.22 ± 1.87	5.78 ± 1.73	<.001
Platelets, 10^3^/cm	204.02 ± 74.08	194.71 ± 72.91	212.37 ± 74.13	<.001
Haemoglobin, g/dl	13.71 ± 1.95	14.62 ± 1.82	12.90 ± 1.70	<.001
Fasting glucose, mg/dl	103.78 ± 35.49	103.79 ± 35.45	103.77 ± 35.53	.328
LDL cholesterol, mg/dl	102.32 ± 29.03	98.67 ± 28.31	105.60±29.29	<.001
HDL cholesterol, mg/dl	51.18 ± 11.59	49.97 ± 12.35	52.27 ± 10.75	<.001
Total cholesterol, mg/dl	184.10 ± 36.43	177.54 ± 35.37	189.98 ± 36.37	<.001
Glycated haemoglobin (HAb1C), %	5.98 ± 0.99	5.94 ± 0.94	6.03 ± 1.04	.001

### 3.2 Prevalence of pre‐diabetes and diabetes

**FIGURE 1:**
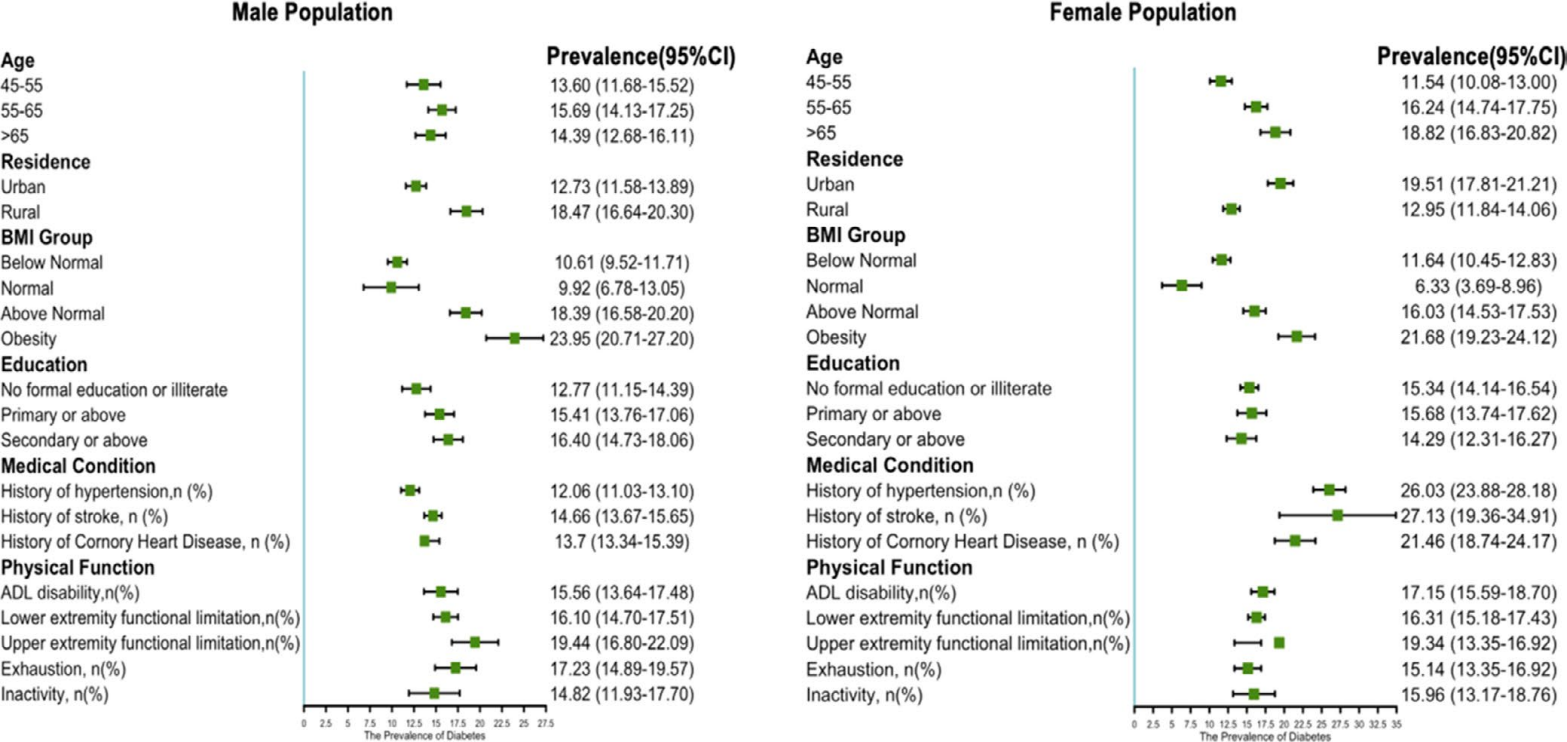
Prevalence of diabetes among male and female population

Prevalence rates of pre‐diabetes and diabetes in different subgroups of participants are shown in Table 2 alongside 95% confidence limits. The overall unadjusted prevalence of diabetes was 13.21% (95% CI: 12.62%–13.81%), and the overall prevalence of pre‐diabetes was 25.16% (95% CI: 24.39%–25.92%) in this cohort. Prevalence of diabetes is 12.37% in the 45 ~ 55 year group and increases with age, and more than one quarter of respondents (26.45%) have pre‐diabetes among those aged between 55 and 65 years. There were no differences in the prevalence of diabetes and pre‐diabetes among different sex and participants with different educational background. The overall standardized prevalence of diabetes adjusted for age and sex was 14.76%, and 12.59%, 15.96% and 16.69% among participants aged between 45 ~ 55 year, 55 ~ 65 year and over 65 years, respectively. Higher prevalence of diabetes and pre‐diabetes is found among respondents who live in urban areas than those who live in rural area. Prevalence of diabetes and pre‐diabetes increases with BMI, from 11.11% (95% CI: 10.30%–11.91%) in BMI < 18.5 group to 22.54% (95% CI: 20.59%–24.49%) in BMI > = 28 group. There is a higher pre‐diabetes prevalence than diabetes prevalence for those who had a history of hypertension, stroke, coronary heart disease, ADL disability and lower/upper extremity functional limitation (*p *< .001). Figure 1 summarizes diabetes prevalence by sex categories. Moreover, we observed differences in systolic BP, diastolic BP, platelets and fasting glucose between normal, pre‐diabetes and diabetes participants (Table 3).

**TABLE 2:** Rates of pre‐diabetes and diabetes within subgroups of socioeconomic characteristics and health status

Socioeconomic characteristics	*N*	Diabetes prevalence (%, 95% CI)	Pre‐diabetes (%, 95% CI)	*p*‐Value
Age				<.001
45 ~ 55	3065	12.37 (12.20–13.53)	21.60 (20.14–23.06)	
55 ~ 65	4400	15.98 (14.89–17.06)	26.45 (25.15–27.76)	
>65	3089	16.52 (15.20–17.82)	27.29 (25.72–28.86)	
Gender				.79
Male	5889	14.62 (13.2–15.52)	25.61 (24.49–26.72)	
Female	6569	14.71 (13.85–15.56)	24.75 (23.71–25.80)	
Residence				<.001
Urban	3813	19.04 (17.79–20.29)	27.56 (26.14–28.98)	
Rural	6741	12.85 (12.05–13.65)	24.02 (22.98–25.02)	
BMI Group				<.001
Underweight	685	11.11 (10.30–11.91)	22.25 (21.18–23.31)	
Normal	5826	8.18 (6.12–10.23)	17.81 (14.94–20.68)	
Overweight	4058	17.05 (15.90–18.21)	28.43 (27.05–29.83)	
Obesity	1766	22.54 (20.59–24.49)	30.07 (27.93–32.21)	
Education				.337
No formal education or illiterate	5105	14.52 (13.55–15.48)	25.47 (24.27–26.66)	
Primary or above	3195	15.52 (14.27–16.78)	24.91 (23.41–26.41)	
Secondary or above	3107	15.58 (14.30–16.85)	25.56 (24.02–27.09)	
Health Condition				
History of hypertension	2871	25.18 (23.59–26.77)	29.57 (27.90–31.24)	<.001
History of stroke	280	27.86 (22.57–33.14)	25.36 (20.23–30.48)	<.001
History of Coronary Heart Disease	1463	20.92 (18.83–23.00)	26.52 (24.26–28.79)	<.001
Physical Function				
ADL disability	3632	16.55 (15.34–17.76)	25.69 (24.27–27.10)	<.001
Lower extremity functional limitation	6803	16.23 (15.35–17.10)	25.34 (24.31–26.38)	<.001
Upper extremity functional limitation	2529	19.38 (17.83–20.92)	24.28 (22.61–25.95)	<.001
Exhaustion	2550	15.96 (14.54–17.38)	22.82(21.19–24.45)	.102
Inactivity	1251	15.43 (13.42–17.43)	23.34 (20.99–25.69)	.288

**TABLE 3:** Association of clinical measures with diabetes

Clinical Characteristics	Diabetes prevalence (Mean ± SD)	Pre‐diabetes (Mean ± SD)	Normal (Mean ± SD)	*p*‐Value
Systolic pressure (mmHg)	133.02 ± 20.49	130.91 ± 19.23	127.75 ± 19.72	<.001
Diastolic pressure (mmHg)	77.06 ± 9.58	76.54 ± 9.12	75.20 ± 9.15	<.001
WBC count, 10^3^/cm	6.35 ± 2.06	6.09 ± 1.78	5.86 ± 1.76	.999
Platelets, 10^3^/cm	199.48 ± 68.76	205.42 ± 72.15	204.42 ± 75.90	.011
Haemoglobin, g/dl	13.72 ± 1.92	13.78±1.92	13.68 ± 1.97	.555
Fasting glucose, mg/dl	165.53 ± 65.95	108.66 ± 6.73	88.77 ± 8.05	<.001
LDL cholesterol, mg/dl	102.45 ± 30.76	104.96 ± 30.29	101.21 ± 28.05	1.000
HDL cholesterol, mg/dl	47.83 ± 11.07	50.35±11.05	52.23 ± 11.75	.11
Total cholesterol, mg/dl	187.88 ± 39.92	189.13 ± 37.79	181.25 ± 34.77	.427

**FIGURE 2:**
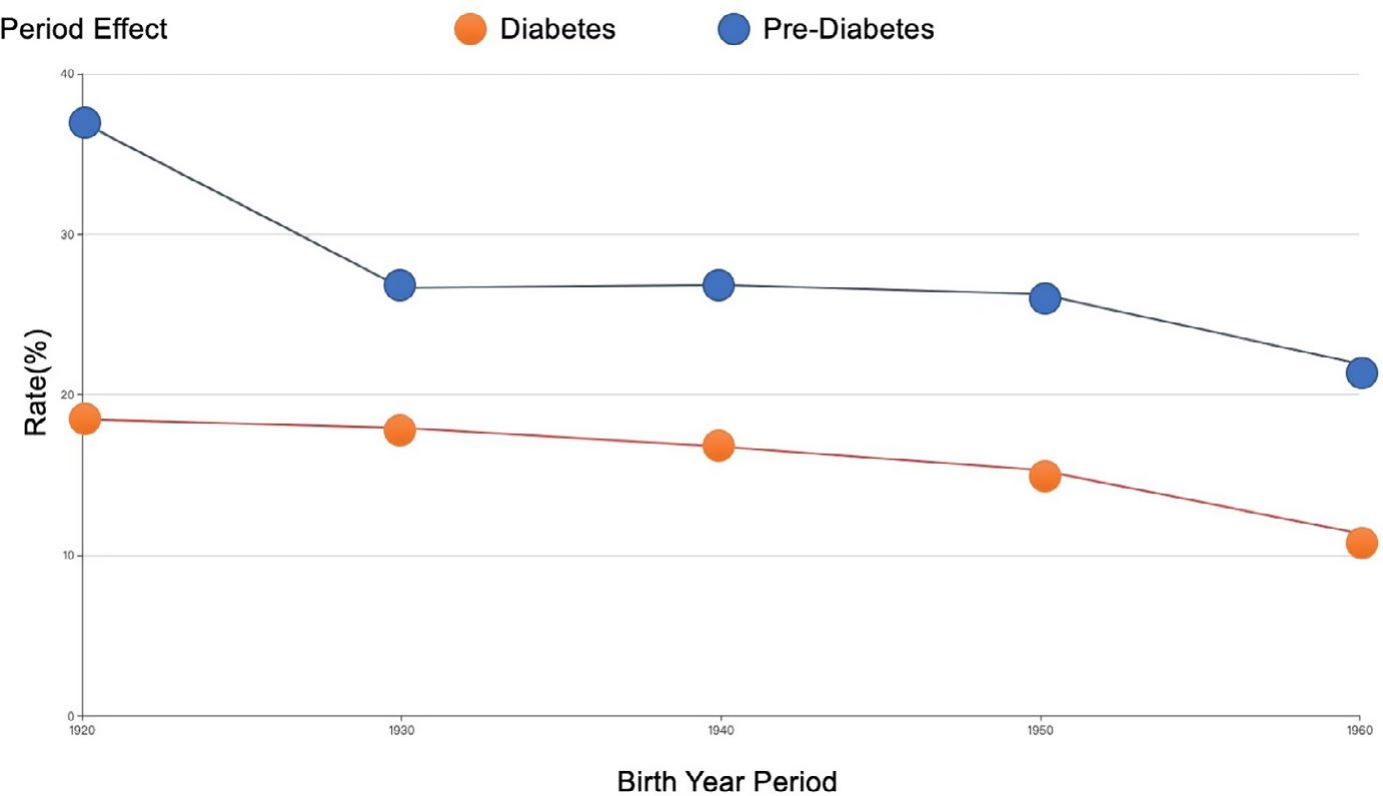
Prevalence of diabetes and pre‐diabetes by age–period–cohort analysis

Figure 2 shows a graphical display of age–period–cohort analyses. We found that the prevalence of diabetes and pre‐diabetes peaked among those who were born in 1920 s, and decreased moderately over the 40‐year period.

### 3.3 Prevalence of awareness, treatment and control among patients with diabetes

Table 4 shows the prevalence rates of awareness, treatment and control in different subgroups of participants. Among people with diabetes, 39.29% (95% CI, 37.06%–41.54%) were aware of their condition: 35.77% (95% CI, 32.56%–38.98%) of men and 42.44% (95% CI, 39.32%–45.57%) of women. Among all patients who were aware of their diabetes, 47.04% (95% CI, 44.86%–49.22%) were treated for this condition: 43.45% (95% CI, 40.27%–46.63%) of men and 50.19% (95% CI, 47.19%–53.18%) in women. Among those patients who were aware of their diabetes, 59.22% (95% CI, 56.97%–61.48%) had their HbA1c values controlled to a concentration of less than 7.0%: 61.44% (95% CI, 58.18%–64.70%) of men and 57.25% (95% CI, 54.12%–60.37%) of women.

**TABLE 4:** Prevalence of awareness, treatment and control among patients with diabetes by different characteristics (*n* = 1587)

Characteristics	Awareness (%, 95% CI)	Treatment (%, 95% CI)	Control (%, 95% CI)	*p*‐Value
Age (years)				.17
45 ~ 55	34.04 (29.24–38.83)	41.06 (36.30–45.82)	59.37 (54.40–64.33)	
55 ~ 65	46.09 (42.39–49.78)	51.57 (48.02–55.12)	58.04 (54.38–61.69)	
>65	44.51 (40.18–48.84)	48.95 (44.83–53.06)	62.94 (58.74–67.15)	
Gender				.021
Male	35.77 (32.56–38.98)	43.45 (40.27–46.63)	61.44 (58.18–64.70)	
Female	42.44 (39.32–45.57)	50.19 (47.19–53.18)	57.25 (54.12–60.37)	
Residence				.11
Rural	39.61 (36.34–42.87)	45.04 (41.90–48.18)	62.70 (59.47–65.93)	
Urban	46.42 (42.78–50.06)	52.18 (48.67–55.69)	56.61 (53.00–60.23)	
BMI Group				<.001
Underweight	38.95 (35.18–42.72)	42.04 (38.36–45.71)	66.77 (63.13–70.41)	
Normal	39.29 (26.09–52.48)	46.27 (34.01–58.52)	69.64 (57.22–82,07)	
Overweight	41.76 (38.08–45.45)	50.39 (46.84–53.95)	53.90 (50.18–57.63)	
Obesity	35.93 (31.20–40.66)	50.11 (45.47–54.75)	54.27 (49.36–59.19)	
Education				.238
No formal education or illiterate	40.62 (37.08–44.17)	47.42 (44.02–50.82)	60.86 (57.34–64.39)	
Primary or above	40.32 (35.99–44.65)	44.57 (40.34–48.80)	62.10 (57.81–66.38)	
Secondary or above	44.63 (40.18–49.07)	51.34 (47.04–55.63)	53.93 (49.47–58.38)	
Health Condition				
History of hypertension	57.82 (54.21–61.42)	58.15 (54.71–61.60)	56.43 (52.81–60.05)	.005
History of stroke	53.84 (42.53–65.16)	62.35 (51.84–72.87)	53.85 (42.53–65.16)	.284
History of Coronary Heart Disease	63.73 (58.31–69.14)	64.86 (59.83–69.88)	57.52 (51.95–63.09)	.002
Physical Function				
ADL disability	45.42 (41.43–49.42)	54.29 (50.56–58.03)	57.90 (53.94–61.86)	.003
Lower extremity functional limitation	44.66 (41.72–47.59)	54.29 (50.56–58.03)	57.43 (54.51–60.35)	<.001
Upper extremity functional limitation	47.55 (43.11–51.99)	56.73 (52.57–60.88)	57.55 (53.16–61.94)	.026
Exhaustion	42.51 (37.68–47.33)	49.56 (44.93–54.18)	59.21 (54.42–64.01)	.787
Inactivity	37.82 (30.92–44.73)	43.93 (37.22–50.63)	56.99 (49.95–64.04)	.89

### 3.4 Factors related with awareness, treatment and control of diabetes

Table 5 presents the results of the multivariate logistic regression analyses. We found that obesity, history of hypertension, history of coronary heart disease and inactivity were significant risk factors of awareness of diabetes; history of hypertension, history of coronary heart disease, ADL disability and lower extremity functional limitation were significantly associated with treatment of diabetes; living in urban area, having lower extremity functional limitation, being overweight and having a history of coronary heart disease were significant risk factors of control of diabetes.

**TABLE 5:** Factors related with awareness, treatment and control of diabetes in the logistic regression models

Characteristics	Awareness		Treatment		Control	
	Adjusted Odds Ratio (95% CI)	*p*	Adjusted Odds Ratio (95% CI)	*p*	Adjusted Odds Ratio (95% CI)	*p*
Age						
45 ~ 55	Reference		Reference		Reference	
55 ~ 65	1.70 (1.13–2.57)	.011	1.44 (0.98–2.11)	.061	0.99 (0.67–1.48)	.975
>65	1.24 (0.78–1.96)	.368	0.99 (0.65–1.52)	.963	1.37 (0.87–2.15)	.17
Gender						
Male	Reference		Reference		Reference	
Female	1.17 (0.83–1.66)	.373	1.07 (0.77–1.49)	.68	0.80 (0.57–1.14)	.215
Residence						
Rural	Reference		Reference		Reference	
Urban	1.28 (0.92–1.77)	.14	1.34 (0.98–1.82)	.064	0.65 (0.47–0.90)	.009
BMI Group	0.00 (0.00–0.00)					
Underweight	0.90 (0.37–2.17)	.806	1.05 (0.47–2.35)	.909	0.97 (0.39–2.41)	.945
Normal	Reference		Reference		Reference	
Overweight	0.95 (0.66–1.37)	.793	1.24 (0.88–1.76)	.233	0.58 (0.40–0.84)	.003
Obesity	0.62 (0.39–0.97)	.036	1.18 (0.78–1.79)	.431	0.84 (0.54–1.29)	.421
Education						
No formal education or illiterate	Reference		Reference		Reference	
Primary or above	1.22 (0.82–1.82)	.321	1.00(0.69–1.44)	.98	0.92 (0.62–1.37)	.691
Secondary or above	1.27 (0.82–1.95)	.288	0.96 (0.63–1.45)	.84	0.87 (0.56–1.33)	.509
Health Condition						
History of hypertension	2.03 (1.45–2.84)	.001	1.85(1.35–1.53)	<.001	0.78 (0.56–1.08)	.138
History of stroke	1.60 (0.76–3.40)	.218	1.80 (0.86–3.82)	.118	0.46 (0.22–1.08)	.138
History of Coronary Heart Disease	2.30 (1.48–3.56)	<.001	1.94 (1.29–2.92)	.002	1.67 (1.07–2.60)	.023
Physical Function						
ADL disability	0.71 (0.47–1.07)	.099	0.65(0.45–0.96)	.028	1.15 (0.77–1.72)	.488
Lower extremity functional limitation	1.33 (0.92–1.92)	.136	1.52 (1.07–2.16)	.019	0.65 (0.45–0.93)	.02
Upper extremity functional limitation	1.06 (0.71–1.58)	.779	1.20 (0.82–1.75)	.35	1.12 (0.75–1.67)	.576
Exhaustion	0.96 (0.66–1.41)	.839	0.83 (0.58–1.19)	.311	1.10 (0.75–1.61)	.621
Inactivity	1.05 (0.14–0.47)	<.001	0.75 (0.52–1.08)	.121	0.94 (0.65–1.38)	.765

## 4 DISCUSSION

In this large, nationally representative sample of Chinese adults aged 45 years or older, we found the prevalence of diabetes among Chinese adults aged 45 years or older in 2015 was 13.21% (95% CI: 12.62%–13.81%), and the overall prevalence of pre‐diabetes was 25.16% (95% CI: 24.39%–25.92%). Compared to research in other countries (7.5% in Canada^10^; 7.95% in Lebanon^11^; 3.12% in Russia^12^; etc.), the prevalence of diabetes was higher in China. All the data indicated that diabetes may have become an urgent problem among Chinese middle‐aged and older adults, and a potential epidemic of diabetes‐related complications is just around the corner. Furthermore, among diabetic patients, only 39.29% were aware of their condition, and among them 47.04% were treated for this condition, which indicated that effective national intervention programmes aimed at discovery and treatment of diabetes should be implemented immediately in China.

The prevalence of diabetes for all age groups worldwide was estimated to be 2.8% in 2000 and 4.4% in 2030.^13^ The prevalence of diabetes in Asian populations has increased rapidly in recent decades with a disproportionate burden among young and middle‐aged individuals, and China is now among the countries with the highest diabetes prevalence in Asia.^3^ The overall prevalence among Chinese adults aged 18 years or older was 11.6% in the adult, 12.1% in men and 11.0% in women.^14^ Another nationally representative sample of Chinese adults aged 20 years or older showed that the prevalence of total diabetes and pre‐diabetes was 9.7% (10.6% among men and 8.8% among women) and 15.5% (16.1% among men and 14.9% among women),^15^ respectively. In one representative sample of noninstitutionalized Korean adults, the crude prevalence of diabetes in adults aged ≥30 years increased from 10.0% to 12.7% from 2007 to 2017.^16^ Similarly, in this study, we found the prevalence of diabetes among Chinese adults aged 45 years or older in 2015 was 13.21% (13.6% in male and 11.54% in female), and the overall prevalence of pre‐diabetes was 25.16%. The prevalence of diabetes in our present study was also relatively higher than in some regional surveys in China reported in recent years, such as in Kazakh adults in Xin Jiang (7.3%, 11.6% and 10.5% in the age groups of 45–54, 55–64 and 65 years or older, respectively).^15^ However, the prevalence of diabetes among Chinese rural population aged 40 years and older was higher, climbing to 24.7%.^9^ The difference might be due to the discrepancy in the socioeconomic status of different region, as well as age, lifestyles and dietary habits among different population.^9^

In our study, we also found that age, residence, BMI, history of hypertension, stroke and coronary heart disease, having ADL disability and lower/upper extremity functional limitation were significantly associated with higher risk of diabetes and pre‐diabetes. The prevalence of diabetes was higher in male population aged 45 ~ 55 years than in female. In other age groups, the prevalence of female was greater. It is said that more women are overweight or obese after the age of 45 years, whereas more males are overweight at younger age.^17^ Because obesity is the major risk factor of diabetes in both sexes, the prevalence of diabetes among male in all age groups might be higher than female, and the prevalence of diabetes among female at middle‐aged and older might be greater. It is not surprising that the prevalence of patterns of diabetes across regions resembles those of obesity.^18^ Higher prevalence of diabetes and pre‐diabetes among respondents who live in urban areas than those who live in rural area were observed in this study, which was consistent with previous findings.^5^, ^19^, ^20^ Also, higher prevalence of diabetes and pre‐diabetes was seen among participants with higher BMI, and both obesity and inactivity were strongly associated with decreased odds of awareness of diabetes among diabetic participants. Urbanization is associated with changes in lifestyle that lead to physical inactivity, an unhealthy diet, and obesity, all of which have been implicated as contributing factors in the development of diabetes.^15^ Participants living in urban areas were also more likely to be well informed and report antidiabetic treatment and to control their diabetes better than rural residents.^21^ These may reflect the problem of accessibility and the quality of health care among this disadvantaged group. The government should put into place basic medical and health care covering more rural residents in the future health policy setting and ensure more rural resident have access to convenient and affordable basic medical and health services.

Additionally, we observed significant differences in systolic BP, diastolic BP, platelets and fasting glucose between normal among pre‐diabetes and diabetes participants. Since the functional changes occurring in diabetes and hypertensive conditions significantly alter the haemodynamic stress on the heart and other organs, prior study found that people with diabetes and hypertension were associated with an increased risk of cardiovascular mortality compared with those with either condition alone.^22^ Several pathogenic mechanisms have been proposed to explain the association between diabetes and hypertension,^23^ including the incretin‐mediated control of the renin–angiotensin–aldosterone system and alterations in calcium–calmodulin system. Therefore, the blood pressure and biomarkers of diabetic participants need to be closely monitored to reduce the mortality caused by cardiovascular disease among middle‐aged and older Chinese adults.

Findings from our survey also showed that the overall proportion of patients who were aware of their diabetes condition was 39.29%; of those who were aware of their condition, 47.04% were receiving antidiabetic medication, which were lower than the results in developed countries, such as Latin America (79.8% awareness rate, 58.8% treatment rate),^24^ Malaysia (65.2% awareness rate, 87.5% treatment rate) ^25^ and Switzerland (65.3% awareness rate, 86.0% treatment rate).^26^ The results is consistent with previous studies in China: rate of treatment was higher than previous study on population aged 18 years and over (25.8%),^3^ and similar to study on adults aged 40 years or older in Jilin Province, Northeast China (47.7%).^27^ Additionally, we found that aged between 55 and 65 years, being obesity, having a history of hypertension and coronary heart disease, as well as being inactivity were both significantly associated with higher odds of awareness, which indicated that individual consciousness played an important role in the awareness of diabetes. The awareness and treatment rates of diabetes in China still need to improve in the future, requiring combined efforts of public health practitioners and professionals and the improvement of people's health awareness. Moreover, among diagnosed diabetes patients, 59.22% had their HbA1c levels controlled. We also observed that living in urban area was significantly associated with higher odds of control among diabetic individuals, which reflected that urban residents could receive more support both emotionally and financially in the treatment and management of their chronic diseases. Therefore, in the future, strategies for increasing socioeconomic status such as increasing income or improved accessibility to good quality of health service may be practicable ways to improve the ability and awareness of diabetes treatment among middle‐aged and elderly adults in China.

The present study had several strengths. First, this is the first study combining information on fasting plasma glucose and other biomarkers, as well as self‐reported diabetic condition to examine the prevalence of diabetes, pre‐diabetes, awareness, treatment and control in a nationally representative sample of noninstitutionalized Chinese adults aged 45 years and older. Second, a multi‐stage stratified random cluster method was used in the sampling process, and complex weighted computation was used in data analysis. Third, venous blood samples were collected and transported to the same laboratory for testing using the same standardized protocol. All these features enhanced the accuracy of prevalence estimates.

Our study also had some limitations. First, we did not distinguish between type 1 and type 2 diabetes among these patients. Second, our study findings are likely to have been influenced by additional factors that were not performed in participants of CHARLS, such as 2‐h oral glucose‐tolerance. Therefore, the prevalence of diabetes and pre‐diabetics was probably underestimated. Finally, because our study was a cross‐sectional survey, we could not establish cause‐and‐effect relationships between the observed associations.

In summary, our study showed that diabetes and pre‐diabetes were highly prevalent among adults aged 45 years or older in China. More troublesome is the finding that awareness, treatment and control rates of diabetes remained relatively low compared with those of developed countries. Given the paucity of data on diabetes among Chinese middle‐aged and older adults, which represent the world's largest ageing population, our study may serve as a basis for future research aimed at identifying physiological, behavioural, and psychosocial risk factors of diabetes, and finally improve the prevention, detection and treatment rates of diabetes in China.

## CONFLICT OF INTEREST

The authors declare no potential conflicts of interest.

## AUTHOR CONTRIBUTIONS

Anying Bai collected data, conceptualized the study, performed statistical analysis and wrote the manuscripts; Jing Tao conceptualized the study and reviewed/edited the manuscripts; Liyuan Tao contributed to the discussion and reviewed/edited the manuscripts; Jue Liu conceptualized the study, acquired the funding, administrated the project and reviewed/edited the manuscript.

## Data Availability

The CHARLS data are publicly available at http://charls.pku.edu.cn.
